# Poor eyesight reveals a new vision gene

**DOI:** 10.7554/eLife.81520

**Published:** 2022-08-05

**Authors:** Tathagata Biswas, Jaya Krishnan, Nicolas Rohner

**Affiliations:** 1 Stowers Institute for Medical Research Kansas City United States; 2 Department of Molecular and Integrative Physiology, University of Kansas Medical Center Kansas City United States

**Keywords:** convergent gene loss, visual acuity, vertebrate evolution, serine proteinase inhibitor, evolution, Mouse, Zebrafish

## Abstract

Comparing the genomes of mammals which evolved to have poor vision identifies an important gene for eyesight.

**Related research article** Indrischek H, Hammer J, Machate A, Hecker N, Kirilenko B, Roscito J, Hans S, Norden C, Brand M, Hiller M. 2022. Vision-related convergent gene losses reveal SERPINE3’s unknown role in the eye. *eLife* **11**:e77999. doi: 10.7554/eLife.77999.

With hundreds of cell types smoothly working together to form clear images of the world, the vertebrate eye can put the most sophisticated digital cameras to shame. Yet many of the genes which establish and maintain this delicate machine remain unknown.

Most mammals have good vision, yet some species have naturally evolved poor eyesight: mice and rats, for instance, have very poor eyesight, while species like the naked mole rat have lost their vision entirely. One way to identify the genetic sequences important for vision is to compare the genomes of species with contrasting visual capacities. Now, in eLife, Michael Hiller (Senckenberg Research Institute), Michael Brand (TU Dresden) and colleagues – including Henrike Indrischek (Max Planck Institute for Molecular Cell Biology and Genetics) as first author – report that a largely uncharacterized gene called *Serpine3* is inactivated in many animals with poor or compromised vision, suggesting it may play an important role in the eye (Indrischek et al., 2022).

First, the team screened the genomes of 49 mammalian species for mutations associated with a severe loss in eye function. This sample included ten species which had poor visual capacity, such as rodents, moles and echolocating bats. A gene was classified as playing a role in eyesight if mutations stopped it from working in more than three species with poor vision. This led to the identification of 29 genes, 15 of which had not been linked to eye development or function before. However, poor vision is mostly restricted to mammals living in low-light habitats which are often limited in nutrients and biodiversity (Olsen et al., 2021). As such, the loss-of-function mutations detected by Indrischek et al. may be unrelated to vision and instead be the result of animals adapting to these challenging environments.

Indrischek et al. then focused on one gene, *Serpine3*, which was predicted to be inactive in seven out of the ten low-vision species (Figure 1). Conversely, animals with excellent vision, such as elephants and chimpanzees, have intact *Serpine3* coding regions (Figure 1). To strengthen their hypothesis, Indrischek et al. added 381 other species with varying visual capabilities to their analysis. Out of the 430 species studied, 70 with poor eyesight had inactivated *Serpine3*.

**Figure 1.**
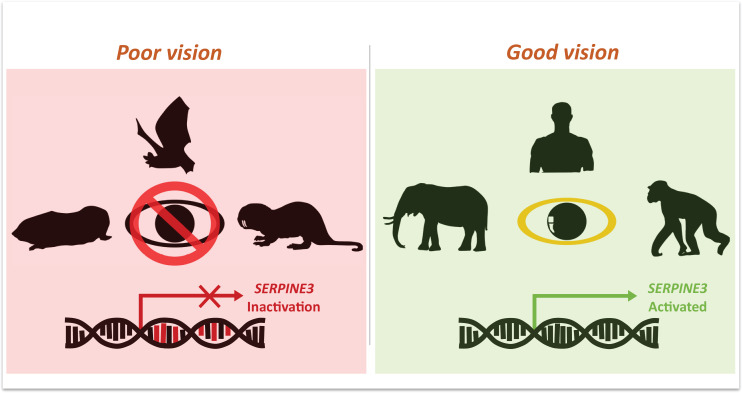
Mutations in *Serpine3* are associated with vision loss. To identify genes that shape the eyes of vertebrates, Indrischek et al. screened the genome of mammals with poor (left, red) and good (right, green) vision. Most animals with poor eyesight – such as cape-golden moles, bats and naked mole rats – had mutations in the gene *Serpine3* which led to its inactivation. However, in mammals with better vision – such as elephants, humans and chimpanzees – the coding region for *Serpine3* was intact and the gene was active. Further experiments confirmed that the product of the *Serpine3* gene is important for good vision.

The product of the *Serpine3* gene belongs to a family of proteins secreted into the extracellular space and implicated in blood clotting, neuroprotection and some human diseases (Law et al., 2006; Barnstable and Tombran-Tink, 2004). To better understand the role *Serpine3* plays in vision, the team carried out functional experiments in zebrafish, as their retinas are organized into layers which are similar to those found in humans (Fadool, 2003). This showed that *Serpine3* is highly expressed in the zebrafish eye, particularly in the inner nuclear layer of the retina. Next, Indrischek et al. deleted *Serpine3* during zebrafish development, causing adult animals to have deformed eyes and disrupting the organization of the cell layers in the retina. This suggests that vertebrate eyes need the product of *Serpine3* in order to function properly.

Finally, Indrischek et al. analyzed a human dataset of genomic sequences from patients with eye-related diseases. They found mutations near to the transcription start site for *Serpine3* are associated with refractive errors in the eye and macular degeneration, suggesting that this gene may also play a role in human eye diseases.

Taken together, these findings suggest that *Serpine3* is important for good vision. In the future, it would be interesting to explore whether the gene is primarily important during development, or to maintain retinal cells and layers during adulthood. This knowledge could help identify new therapies for debilitating eye diseases associated with *Serpine3* or other related genes.

More importantly, Indrischek et al. elegantly demonstrate how studying natural variation in traits such as eyesight can identify the function of uncharacterized genes and the role they may play in disease. Nature is full of characteristics which converged between species over the course of evolution (Farhat et al., 2013; Bergmann and Morinaga, 2019). Applying a comparative genomic approach similar to the one used in this study could transform our understanding of a multitude of other biological processes (Liu et al., 2021; Valenzano et al., 2015; Rohner, 2018). We just have to look.
